# Reducing depressive symptomatology with a smartphone app: study protocol for a randomized, placebo-controlled trial

**DOI:** 10.1186/s13063-017-1960-1

**Published:** 2017-05-12

**Authors:** Cezar Giosan, Oana Cobeanu, Cristina Mogoaşe, Aurora Szentagotai, Vlad Mureşan, Rareș Boian

**Affiliations:** 1 Department of Clinical Psychology and Psychotherapy, Babeș-Bolyai University, Cluj-Napoca, Romania; 2 Berkeley College, New York, NY USA; 3 Department of Psychology, University of Bucharest, Bucharest, Romania

**Keywords:** Depression, Protocol, Randomized trial, Placebo, CBT, Smartphone app

## Abstract

**Background:**

Depression has become one of the leading contributors to the global disease burden. Evidence-based treatments for depression are available, but access to them is still limited in some instances. As technology has become more integrated into mental health care, computerized cognitive behavioral therapy (CBT) protocols have become available and have been recently transposed to mobile environments (e.g., smartphones) in the form of “apps.” Preliminary research on some depression apps has shown promising results in reducing subthreshold or mild to moderate depressive symptoms. However, this small number of studies reports a low statistical power and they have not yet been replicated. Moreover, none of them included an active placebo comparison group. This is problematic, as a “digital placebo effect” may explain some of the positive effects documented until now. The aim of this study is to test a newly developed mobile app firmly grounded in the CBT theory of depression to determine whether this app is clinically useful in decreasing moderate depressive symptoms when compared with an active placebo. Additionally, we are interested in the app’s effect on emotional wellbeing and depressogenic cognitions.

**Methods/design:**

Romanian-speaking adults (18 years and older) with access to a computer and the Internet and owning a smartphone are included in the study. A randomized, three-arm clinical trial is being conducted (i.e., active intervention, placebo intervention and delayed intervention). Two hundred and twenty participants with moderate depressive symptoms (i.e., obtaining scores >9 and ≤16 on the Patient Health Questionnaire, PHQ-9) will be randomized to the three conditions. Participants undergoing therapy, presenting serious mental health problems, or legal or health issues that would prevent them from using the app, as well as participants reporting suicidal ideation are excluded. Participants randomized to the active and placebo interventions will use the smartphone app for 6 weeks. A short therapist check-in via phone will take place every week. Participants in the delayed-intervention condition will be given access to the app after 6 weeks from randomization. The primary outcome is the level of depressive symptomatology. The intervention delivered through the app to the active condition includes psychoeducational materials and exercises based on CBT for depression, while the placebo intervention uses a sham version of the app (i.e., similar structure of courses and exercises).

**Discussion:**

To our knowledge, this study protocol is the first to test the efficacy of a smartphone app for depressive symptomatology in the form of a randomized controlled trial (RCT) that includes an active placebo condition. As such, this can substantially add to the body of evidence supporting the use of apps designed to decrease depression.

**Trial registration:**

ClinicalTrials.gov, identifier: NCT03060200. Registered on 1 February 2017. The first participant was enrolled on 17 February 2017.

**Electronic supplementary material:**

The online version of this article (doi:10.1186/s13063-017-1960-1) contains supplementary material, which is available to authorized users.

## Background

Depression is a highly prevalent and burdensome condition worldwide. According to the World Health Organization, by 2030 depression will be the main contributor to the global disease burden (GDB) [1].

While effective, evidence-based treatments for depression do exist, they are sometimes not accessed appropriately due to a variety of reasons. In fact, fewer than half of those in need of treatment (and in many countries, fewer than 10%) receive adequate care [2]. Barriers to treatment access include stigma, logistical costs, or the limited availability of adequately trained therapists [3, 4]. In this context, increasing treatment accessibility for people suffering from depression is critical.

As technology has become more integrated into mental health care [5, 6] and Internet access has increased [7], computerized treatment protocols have been proposed as potential solutions to overcome the issue of treatment accessibility [8–11]. Cognitive behavioral therapy (CBT), one of the best empirically supported psychological treatments for depression [12], has been adapted for online formats, and research has shown that computerized CBT protocols for depression could yield results comparable to face-to-face CBT in both adult and youth samples [13, 14]. Furthermore, computerized CBT programs for depression show a higher moderate effect size compared to control conditions, with even better results in the case of assisted programs [15]. Yet, many studies testing computerized CBT for depression are of low quality, few included an active placebo group, and even fewer have investigated the mechanisms of change [16]. Moreover, the benefits of treatment have been reported to be rather short lived, the impact on functioning was found to be minimal, and high attrition rates seriously limited the potential clinical benefits [17]. However, in the rush to develop highly accessible, attractive and flexible technological solutions for mental health problems, the problems associated with computerized CBT for depression have been largely ignored.

### Framework and rationale of the present study

In the recent years, some computerized CBT protocols have transitioned to mobile environments (e.g., smartphones) in the form of “apps.” These apps have been designed to allow an even more flexible and personalized treatment for depression. However, since very few of them have ever been tested in randomized clinical trials [18] it is largely unclear if they are beneficial, or, perhaps more importantly, if they might have unintended side effects. Some of these apps claim to provide elements congruent to the best practice guidelines in the field (e.g., being CBT-inspired), but they vary widely in scope, content, and complexity. To our knowledge, none has provided comprehensive evidence-based support for their efficacy.

Several preliminary studies on some depression apps have shown promising results in reducing subthreshold or mild to moderate depressive symptoms [19–24]. Still, such studies are small (pilot studies), too few, and not replicated [18]. Moreover, none of them included an active placebo comparison group. This is problematic, as a “digital placebo effect” may explain – at least partially – some of the positive effects documented by the few existing studies [18]. Such placebo effects could be the result of a variety of factors such as cognitive distraction, behavioral activation (BA), positive expectations (demand effects), the perception of the smartphone as being an extension of self (which could result in feeling better just because the person receives messages and advice through a familiar, personal device; [18] or a combination of these.

In short, the evidence supporting the use of apps designed to decrease depression is mounting, but consistent, additional, experimentally sound work is needed before we can draw clear conclusions.

### Objectives

To discern between a genuine clinical effect and a placebo effect, we need to compare outcomes from participants using an actual app with those from participants using a sham version of it. The present study has been designed specifically to achieve this desiderate. We aim to test a newly developed mobile app firmly grounded in the CBT theory of depression to determine whether this app is clinically useful in decreasing moderate depressive symptoms when compared with an active placebo. Additionally, we are interested in the app’s potential to contribute to the reduction of general negative affect, increasing positive affect, and boosting satisfaction with life. Last, but not least, we aim to verify whether the usage of the tested app can modify depressogenic cognitions.

## Methods/design

### Trial design

This study is a randomized, placebo-controlled trial examining the effects of an mHealth CBT intervention in reducing depressive symptomatology for people with moderate symptoms. Participants are randomly assigned to one of the three arms of the trial: (1) mHealth CBT intervention, (2) placebo intervention (PI), and (3) delayed intervention (wait-list; WL). Major assessments are at baseline, mid-intervention, post intervention, and 3-month follow-up. The participants randomized to the PI and WL trial arms are offered access to the mHealth app upon the completion of the study.

The Consolidated Standards of Reporting Trials (CONSORT) guidelines [25] and the Standard Protocol Items: Recommendations for Interventional Trials (SPIRIT) Statement 2013 [26] are followed (see also Additional file 1: SPIRIT 2013 Checklist: recommended items to address in a clinical trial protocol and related documents).

Participants are blinded to the nature of the placebo intervention, but they are informed that they have a 1 in 3 chance of being assigned to the PI group.

### Generic description of the tested application

DCombat is an innovative smartphone app that uses the basic principles of CBT in treating depressive symptoms. It is available for download for iOS and Android and has the following features:

1. Profile and overview section: following the login, the first screen of the application (“profile” or homepage) offers the user the possibility of choosing and personalizing an avatar. On subsequent occasions, at every login, this first screen summarizes information about the user’s activity and therapeutic progress in a simple and attractive format: the user can check their current “energy level” reached from using the app, their progress through its contents. The app incorporates an attractive design in both the visual presentation and flow of activities [27, 28]. Borrowing from gamification strategies [29], the app integrates two graphical representations similar to role-play games: “Energy” and “Level.” Energy represents a percentage value that decreases over time if the app is not used. The objective is to keep the energy level as close to 100% as possible. Reading articles, completing CBT-inspired exercises and filling out questionnaires are all rewarded with an energy boost. Level represents the user’s progress through the educational articles – reading each article increases the level by 1. The goal is to reach the maximum level (i.e., level 12). The overview provided on the first screen also includes a chart representing the user’s progress in improving their mood over time, measured via a scale in the Evaluation section. The overview screen also provides tasks (similar to homework assigned used in face-to-face therapy) in the form of “missions” that are to be completed by the user. Over the course of the program different missions encourage the users to read articles, fill out questionnaires, plan daily activities, and practice rational thinking by doing the existing exercises. When completing missions users are awarded with “achievements” that provide positive feedback. Achievements are always presented on the profile screen to further incentivize and reward consistent app usage. (see Fig. 1 below, for an example of the profile and overview section)

**Fig. 1:**
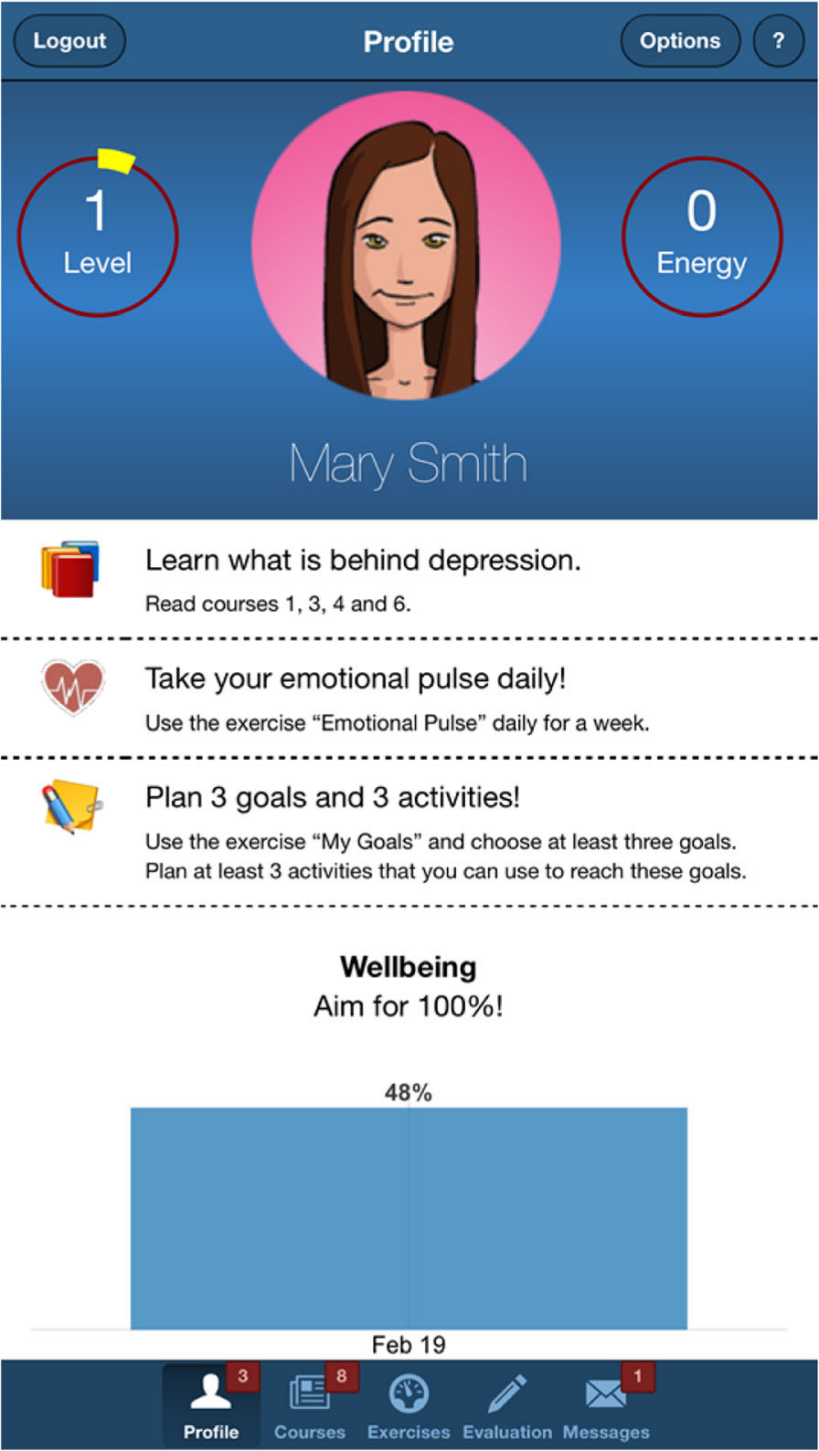
App screenshot with the “profile” and overview section
2. Psychoeducation section: this section contains articles and self-help materials covering topics such as the causes of depression, the impact of dysfunctional thinking patterns and unproductive behaviors, healthy and unhealthy negative emotions, as well as a series of educational videos about relaxation techniques
3. Exercises: the app contains a series of guided cognitive restructuring and behavioral activation (BA) exercises, following the ABCDE model of emotional disturbance [30, 31], and the Brief Behavioral Activation Treatment for Depression described by Lejuez, Hopko, and Hopko [32], respectively. The cognitive restructuring exercises are based on a model where each emotion is assigned to several activating events and the specific underlying rational/irrational beliefs – so that the user is automatically guided through rational thinking patterns without the need for therapist feedback. The BA exercises allow the user to insert content in specific exercise tabs designed around goals, and pleasant activities to be used in the pursuit of these goals (see Fig. 2 for an overview of the “Exercises” section). Completing each activity and exercise is rewarded graphically through a notification and an increase of the Energy percentage (see Fig. 3 below, for an example of the Exercises section – cognitive restructuring exercise)

**Fig. 2:**
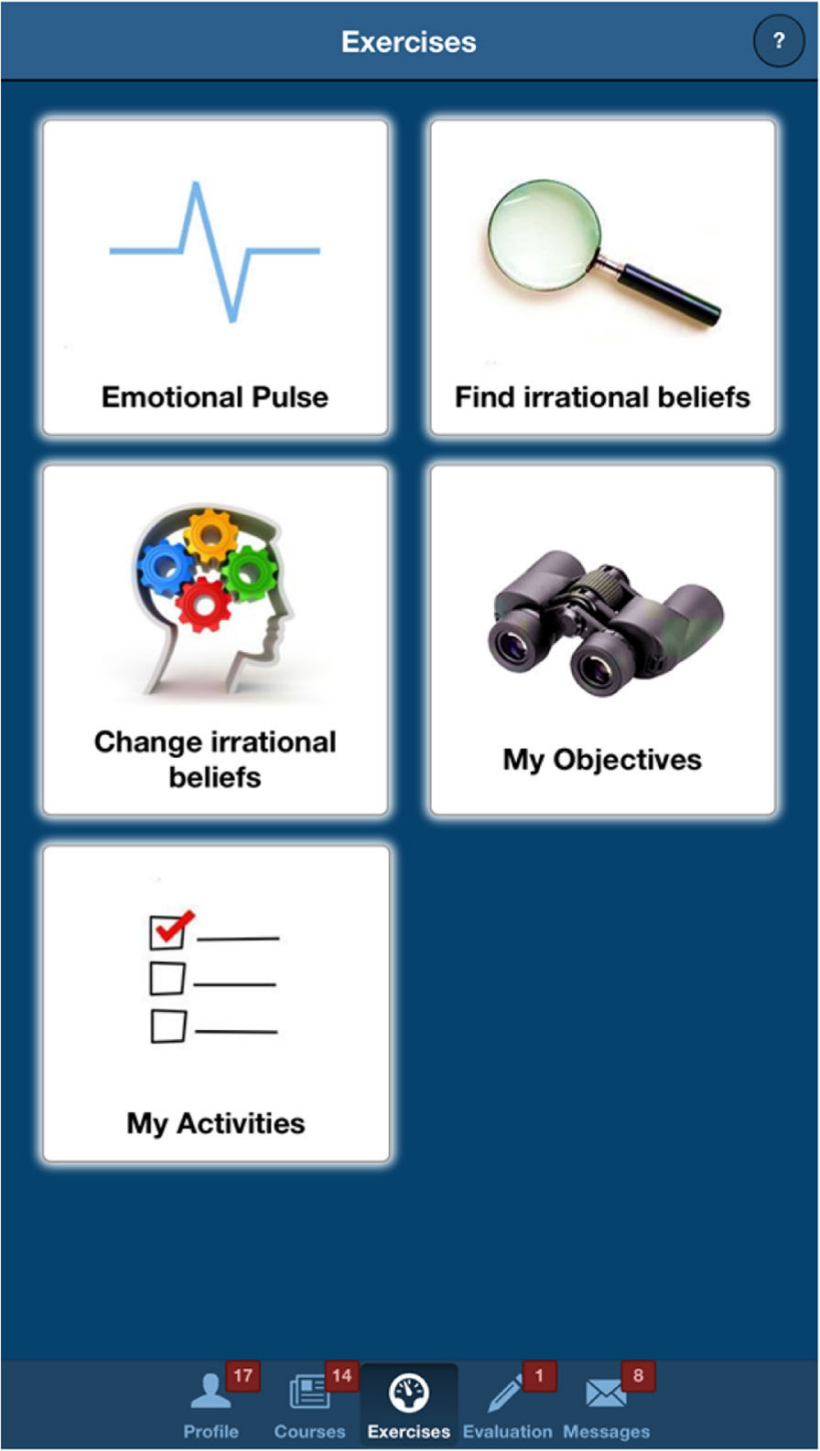
App screenshot of the “Exercises” section

**Fig. 3:**
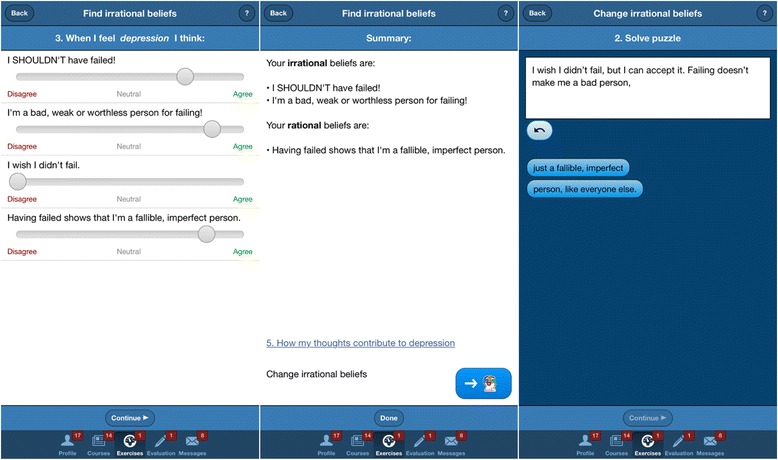
App screenshot of a cognitive restructuring exercise sequence
4. Evaluation section: within the app, the user can fill out questionnaires related to the study being conducted which include a mood inventory. The mood inventory updates the progress graph on the profile section, described earlier. Whenever a new evaluation is due, the user is notified and rewarded with more Energy after completing it.
5. “Messages” section: each week the users are encouraged, via automated messages, to read articles and use the exercises available. They are also reminded to complete that week’s assessment. Moreover, this section allows them to send messages to the research team regarding technical difficulties or other obstacles encountered in using the application’s interface. The user can also use this feature for clinical support or guidance.

### Study setting

The study population consists of Romanian adults with moderate depressive symptomatology. The study is being implemented through the Babeș-Bolyai University, Cluj-Napoca, Romania.

#### Eligibility criteria

##### Inclusion criteria

Healthy Romanian-speaking adults (18 years or older), with access to a computer, a smartphone (Android or iOS), and the Internet are included in the study. The Structured Clinical Interview for *Diagnostic and Statistical Manual of Mental Disorders, Fourth Edition, Text Revision* (DSM-IV) (SCID) [33], specifically, the Overview Module will be used to determine the general clinical status of the participants. A clinical psychologist will conduct a short interview aimed to screen for exclusion criteria (see below). Information on drug use and physical and psychological treatment status will be collected. In addition, to be included in the study, participants should report a Patient Health Questionnaire-9 (PHQ-9) score above 9, but no greater than 16.

##### Exclusion criteria

Participants undergoing therapy (i.e., medication and/or psychotherapy), or presenting substance abuse problems, psychotic symptoms, organic brain disorders (e.g., dementia), self-injury or harming others, or serious health issues that would prevent from using the app, as well as participants reporting scores greater than 1 to question 9 (suicidal ideation) on the PHQ-9 [34] are excluded.

### Study conditions

#### The mHealth intervention

The mHealth CBT intervention comprises the following components: four online intervention modules with specific courses and exercises, activated gradually, a specific assignment for each module, regular automatic messages and assessments, and a therapist check-in per each module.

1. *Courses* represent the psychoeducational and therapeutic background of the intervention, and include the following topics: information on depression, psychological vulnerability, what CBT is, healthy and unhealthy negative emotions, how behaviors contribute to depression, psychological vulnerability, rational and irrational thoughts, treatment adherence, suicidal thoughts, sleep hygiene, social support, relaxation techniques, and relapse prevention
2. *Exercises* use the information presented in the courses and follow the structure of a regular therapy session and/or therapeutic homework

The following exercises are included in the app:

1. The *Emotional Pulse* exercise registers the user’s current activity and emotions (healthy or unhealthy)
2. The BA exercises – “My Goals” and “My Activities,” consisting of setting goals and building a list of rewards/pleasant activities to be used in the pursuit of these goals
3. The *Find Irrational Thoughts* exercise helps in identifying thoughts behind the emotions found with the Emotional Pulse exercise, and
4. The *Change Irrational Thoughts* exercise challenges negative/irrational thinking behind unhealthy emotions and replaces it with healthy, flexible, and functional thoughts

Module 1 of the intervention presents information on depression, introduces the user to the CBT approach, and discusses healthy and unhealthy emotions and behaviors. It also introduces the Emotional Pulse and My Goals exercises. Information on suicidal thoughts and treatment adherence is also available.

Module 2 presents information on psychological vulnerability and rational and irrational beliefs, while introducing the Find Irrational Thoughts and Change Irrational Thoughts exercises.

Module 3 presents information on relaxation techniques, also providing video support, along with information on sleep hygiene and social support.

Module 4 focuses on relapse prevention and continued practice of all the exercises.

Participants are informed that they should complete one module every 10 days, within 6 weeks.

### Therapist check-in

Three clinical psychologists provide all clinical contact with the active and placebo intervention participants, via phone calls. Every call is audiorecorded with the participant’s consent. The total therapist time spent per participant is also recorded. The therapists are instructed to limit contact time to approximately 5 min per participant, unless more time is clinically indicated. The therapist check-in component has the role of monitoring app use and mood status, without engaging in therapy, but encouraging intervention adherence.

#### The placebo control group

The active placebo intervention will be delivered via the same platform and largely in the same format as the tested app: it will include largely the same sections and features as the original app (i.e., profile, courses, exercises, evaluation, and messages). The psychoeducation section, although mirroring the structure of the corresponding section in the original app, will include different content, elaborating on common sense strategies of overcoming depression (like positive thinking, listening to music, etc.) and general knowledge about mental and physical health-relevant topics. A Sticky Notes tab will be available in the exercises section, giving the user the possibility of adding content, while suggesting two possible topics: favorite quotes, and a reading journal.

#### The wait-list control group

Participants in the delayed-intervention group are placed on a wait-list for 6 weeks; then they are given full access to the application.

### Outcomes and measures

#### Primary outcomes

The level of depressive symptomatology constitutes the primary outcome.

#### Secondary outcomes

General positive and negative affect and satisfaction with life constitute the secondary outcomes which have been included to assess the efficacy of the application beyond its impact on specific clinical symptoms, e.g., as a possible tool for the improvement of life quality and general wellbeing.

#### Other outcomes

Dysfunctional cognitions, irrational beliefs, and negative automatic thoughts (conceptualized as mechanisms of change) and BA and/or avoidance will be considered as possible predictors of the outcomes. Satisfaction with the application and data regarding the app usage are also assessed.

The instruments used for each of the abovementioned outcomes are presented below:

#### Screening measures

The PHQ-9 [34] is a nine-question instrument designed to correspond to the *Diagnostic and Statistical Manual of Mental Disorder, Fourth Edition, Revised Text* (DSM-IV-TR) [35] diagnostic criteria for major depressive disorder. Respondents rate the items from 0 to 3 according to the frequency of their experience over the previous 2-week period (0 – not at all; 3 – nearly every day). The score can then be interpreted as indicating depression severity (no depression, mild, moderate, moderately severe, or severe depression).

#### Measures of primary, secondary, and other outcomes

The following measures have been adapted into Romanian and successfully used in two previous trials testing another version of the app, and they have shown adequate psychometric properties [36].

The level of depressive symptomatology is assessed with the Center for Epidemiologic Studies Depression Scale–Revised (CESD-R) [37, 38]. The CESD-R is a 20-item self-report instrument, which measures symptoms of depression in nine different groups: sadness (dysphoria), loss of interest (anhedonia), appetite, sleep, thinking/concentration, guilt (worthlessness), tiredness (fatigue), movement (agitation), and suicidal ideation. Participants rate each item on a 5-point Likert scale, from 0 (not at all or less than 1 day) to 4 (nearly every day for 2 weeks) to indicate how they felt or behaved during the last week or so. The total CESD-R score is calculated as a sum of responses to all 20 questions. The CESD-R exhibited good psychometric properties, including high internal consistency, strong factor loadings, and theoretically consistent convergent and divergent validity with anxiety, schizotypy, and positive and negative affect [39].

*The Positive and Negative Affect Scale* (PANAS) [40] is a 20-item self-report questionnaire designed to assess mood. It consists of 10 items that address positive affect (PA) and 10 items that address negative affect (NA). Participants rate each item on a 5-point Likert scale, from 1 (very slightly/not at all) to 5 (extremely) to indicate how they felt during the indicated time frame. The PANAS can be used to assess mood on various time scales by altering the instructions. For the purposes of this study we have used a 2-week time frame. The validity and internal consistency of the PANAS are good, with test-retest reliability being the highest for the “general” temporal instruction. The PANAS has been used previously on the Romanian population and was found to have adequate psychometric properties [41, 42].

*Satisfaction with Life* (SWL) [43] is a five-item scale designed to measure global cognitive judgments of one’s life satisfaction. Participants rate each of the five items using a 7-point scale that ranges from 7 (strongly agree) to 1 (strongly disagree). The SWL has been shown to be a valid and reliable measure of life satisfaction which can be used with a wide range of age groups [44, 45].

*The Dysfunctional Attitudes Scale* (DAS) [46] was designed to measure the intensity of dysfunctional attitudes, which, according to the cognitive theory of depression [47], contribute to vulnerability for depression. For the purposes of our study, we used the short form of this scale. The Dysfunctional Attitudes Scale–Short Form (DAS-SF) [48] consists of two subscales: “dependency” (6 items) and “perfectionism/performance evaluation” (11 items). The 17 items are rated on a 7-point Likert scale, from 1 (total disagreement) to 7 (total agreement). The DAS-SF possesses good psychometric properties in terms of model fit, reliability and convergent construct validity [48].

*The Beliefs Scale* (BS) [49] measures irrational beliefs. It consists of 20 items and responders indicate how much they agree or disagree with each item using a 5-point Likert scale that ranges from 1 (strongly disagree) to 5 (strongly agree). The BS shows good psychometric properties regarding construct and discriminant validity [50].

*The Automatic Thoughts Questionnaire* (ATQ) [51] is a 15-item self-report measure used to assess depression-related cognitions. Participants rate, on a 5-point Likert scale from 1 (never) to 5 (almost all the time), how frequently they have had a given thought over the past week. A higher score shows a higher frequency of automatic thoughts. The psychometric properties of the ATQ have been adequately demonstrated in previous studies [52]. The ATQ has been successfully used previously on the Romanian population [53–55].

*The Behavioral Activation for Depression Scale–Short Form* (BADS-SF) [56] is an instrument designed to be administered weekly to measure changes in avoidance and activation over the course of the BA treatment for depression. The BADS consists of nine items grouped into two subscales (Activation and Avoidance/Rumination). Respondents rate each item on a 7-point Likert scale ranging from 0 (not at all) to 6 (completely). The scale enjoys good psychometric properties [57].

*The Satisfaction with the Application Scale* was specifically designed for this study. It consists of 10 items that assess users’ satisfaction with the application, its difficulty level, attractiveness, and subjective utility. The first eight items are rated on a 3-point scale, ranging from 0 to 2. Each response scale is personalized to the content of the item (e.g., How attractive did you find the exercises included in the application?: 0 = rather unattractive, 1 = attractive enough, 2 = very attractive). Item 9 assesses the application globally, with the participant being asked to give an overall grade between 1 (minimum) and 10 (maximum). Item 10 asks the participants if they would recommend the application to a friend (“yes” or “no” answer).

*The Application Use Scale* was also developed specifically for this study. It consists of eight items that assess weekly quantitative app usage aspects: the effort invested in homework (one item), number of practiced exercises (one item), number of read courses (one item), frequency of general application use (one item), and frequency of every exercise use (four items).

#### Participant timeline

Potential participants will be assessed for eligibility through an initial assessment of depressive symptoms. The initial assessment phase consists of administration of the PHQ-9 and a short phone screening interview. Applicants who do not meet the inclusion criteria are informed via e-mail. They are given a summary score and interpretation for their PHQ-9 score and they are encouraged to discuss their problems with a professional, if necessary. Information on how to reach one – a clinical psychologist or a psychotherapist – is also provided.

**Fig. 4:**
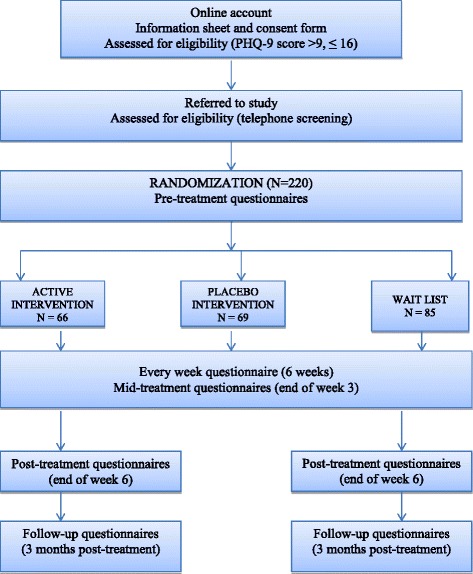
Consolidated Standards of Reporting Trials (CONSORT) flow diagram [25] showing subject allocation to the study conditions

**Fig. 5:**
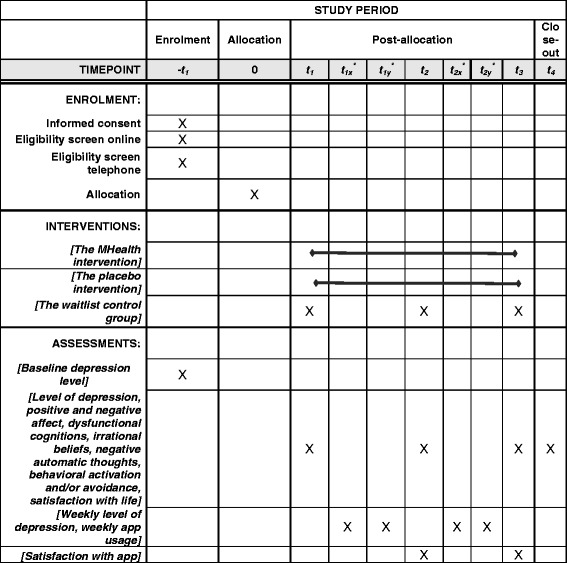
Schedule of enrolment, interventions, and assessments

After the initial assessment, the participants meeting the inclusion criteria will be randomly assigned to one of the three conditions: (1) active intervention condition – mHealth CBT (group 1), (2) the PI (group 2), or (3) the delayed-intervention condition (WL) (group 3) (see flow diagram in Fig. 4). Subsequent assessments consist of all the instruments presented in the “Outcome measures” section. Figure 5 shows the schedule of enrollment, interventions, and assessments which accords with the SPIRIT figure.

The participants in the active intervention and active placebo conditions are assessed at preintervention (time 1 – baseline), at mid-intervention (time 2 – 3 weeks after baseline), at post intervention (time 3 – 6 weeks after baseline), and at 3 months post intervention (time 4). The participants in the delayed-intervention condition are assessed before the waiting period (time 1 – baseline), at mid-waiting period (time 2 – 3 weeks after baseline), and at post waiting period (time 3 – 6 weeks after baseline).

Participants assigned to the active intervention condition will be given access to the online application along with explicit instructions regarding the use of all its sections. Participants will be given 6 weeks to complete the intervention, during which time weekly messages will be sent out to them. Messages include regular assignments designed for a complete and thorough use of the application’s courses and exercises, and encourage the review of materials whenever possible. However, participants are free to use the application at their own pace.

Similarly, the participants in the active placebo condition will be given access to a sham version of the app. During the 6-week intervention period they will be exposed to the same amount of online (i.e., in-app messages) and therapist communication as the active intervention participants.

After each week, participants’ app use is evaluated (see the Application Use Scale described above). Similarly, after each week of using the app, the participants are required to complete the CESD-R to monitor their depressive symptomatology and provide individualized in-app feedback on their emotional state.

The participants in the delayed-intervention condition are placed on a wait-list for 6 weeks, after which time they are given full access to the app.

#### Sample size

A total of 165 participants with moderate depressive symptomatology was estimated. This is based on previous studies on the efficacy of mobile phone applications for depression, which reported small to moderate between-group effect sizes [19, 24]. An a priori sample size calculation was conducted with G*Power 3.1.9.2 [58, 59] for analysis of variance (ANOVA) (repeated measures, between factors), assuming an alpha level of 0.05, a statistical power of 0.80, and a small to medium effect size (*f* = 0.2). Since the current protocol has a therapist check-in component, a sample size recruitment target of *N* = 220 was set to allow for sample attrition, thus estimating a dropout rate of approximately 30%.

### Recruitment

Possible participants are approached through ads on social networks. Those interested in using the application are asked to access the study’s website – www.binedispus.ro, read the information package, and follow the online standard recruitment procedure. They are subsequently contacted by phone, at which point the enrollment procedure is described and the short screening phone interview takes place.

### Assignment to study group

The participants are randomly assigned to one of the three trial arms, using the software Randomizer.org. Randomization is performed by a research assistant using a simple (unrestricted) randomization sequence that assigns three unique numbers per participant; the number assigned is either 1, 2, or 3 according to the number of the experimental condition. To conceal the allocation mechanism, the same research assistant will monitor the assessments and allow access to the application for the participants in the wait-list control group after 6 weeks. The principal investigator and the statisticians running the data analysis will remain blinded to the study condition until the completion of the study.

### Data collection, management, and analyses

#### Data collection methods

The initial assessment data are collected via the project website. Afterwards, a research assistant conducts telephone-screening interviews with potential participants to determine eligibility, and provides clinical screening reports. All subsequent assessment data are collected within the application, in the Evaluation section, and transferred to our servers using an encrypted, secure (https) industry-standard transfer protocol.

As far as participant retention and follow-up completion are concerned, the therapist check-in component of both the active and placebo interventions will play an important role. Besides being reminded of the specific assessment that needs to be completed after each intervention module, through the Messages section, the participants will also be encouraged by their assigned therapist to complete the assessments. Also, the therapist informs them that the weekly completion of the mood questionnaires allows the app to create a personalized profile of their mood, along with individualized visual feedback on their emotional state. Moreover, they are rewarded with more Energy after completing every assessment. Finally, for the follow-up assessments, the participants will receive new messages reminding them that free use of the app was possible because of a research grant, and thus data collection is essential. Additionally, participants are informed that they can attend an online psychological assessment with a clinical psychologist, based on the completion of follow-up questionnaires, if they wish to do so.

#### Statistical methods

Post-treatment analyses involve an intent-to-treat (ITT) design with the first available data after the baseline carried forward.

Improvement in the primary outcomes measures scores within and between the groups will be examined using mixed-effects linear regression analyses with a random intercept and slope over time (four assessments: baseline, mid-intervention, post intervention, and follow-up) and fixed effects for intervention assignment. The main analysis will compare groups (active intervention, PI, and WL) in terms of the level of depressive symptoms. When the analysis of secondary/other outcomes is performed, the error probability will be adjusted according to the number of group comparisons performed.

App usage data and user activity will be examined by comparing, for example, the participants’ self-reported activity with their actual app usage as recorded by our software. This will inform us about any obstacles to user adherence to the protocol, as well as specific usage patterns.

### Monitoring study implementation

Three clinical psychologists, members of the study team, screen for the risk of unintended effects or harm to the participants (i.e., clinically significant increase in depressive symptomatology as measured by the CESD-R). Every participant’s CESD-R score is assessed weekly and interpreted clinically by the clinician assigned to them. If the participant does not complete the CESD-R assessment, the clinician will address this issue during the phone check-in. If necessary, the therapist can recommend the interruption of the participant’s access to the application.

### Ethics and dissemination

The Ethics Commission at Babeș-Bolyai University has approved this study. Although the intervention that it delivers cannot be considered a treatment for depressed people, it does target individuals with moderate depressive symptomatology who may be at risk for developing more severe depressive symptomatology. The screening process and the exclusionary criteria identify people who may be at risk for suicide. These people are immediately referred to the appropriate clinical services. If, during the use of the application, a participant’s condition worsens, the assigned clinician can recommend the interruption of that user’s access to the application. The project supervisor can further decide on such matters and, if necessary, make a further referral or recommendation. As far as data confidentiality is concerned, special efforts were made to secure the data online collection and storage. Both data collection means (i.e., via web browser of the mobile app) communicate with the server over industry-standard secure connections (i.e., https). The server stores the data in a password-protected database that is not publicly accessible. The system running our applications is kept up to date to prevent intrusions. Access to the electronic data is password-protected and only available to the project team of researchers and programmers. The passwords are changed regularly. The clinical screening reports contain no personal identifying information.

### Dissemination policy

Information on the development of the application and its clinical relevance has been presented at several international and national conferences. A first exploratory study protocol on the efficacy of the app in reducing cognitive vulnerability and mild depressive symptoms has already been published [36]. More information regarding research progress and dissemination can be found at the project’s designated website [60]. This trial’s results will be submitted for publication in a peer-reviewed journal, focusing on primary and secondary outcomes results.

## Discussion

This study describes a randomized clinical trial testing a smartphone app aimed at reducing moderate depressive symptoms. The target of this trial represents people with moderate depressive symptomatology. As there is no current clear evidence about the efficacy of smartphone apps in reducing depressive symptomatology, despite some preliminary studies that have shown promising results [19–24], this research can contribute substantially to clarifying the therapeutic merits of mobile interventions.

This study’s main aim is to test a newly developed app based on the CBT theory of depression to determine primarily whether this app is clinically useful in decreasing moderate depressive symptoms when compared with an active placebo. To our knowledge, this is the first attempt to test the efficacy of a smartphone app for depressive symptomatology in the form of an RCT that includes an active placebo condition. As such, this can substantially add to the body of evidence supporting the use of apps designed to decrease depression.

Additionally, this study investigates the app’s potential to contribute to the reduction of general negative affect, increasing positive affect, boosting satisfaction with life, and modifying depressogenic cognitions.

### Trial status

Participant recruitment has begun on 15 February 2017. Randomization of the participants was performed on the same day.

## Additional file

Additional file 1: SPIRIT 2013 Checklist: recommended items to address in a clinical trial protocol and related documents. (DOC 122 kb)

## Abbreviations

ATQ: Automatic Thoughts Questionnaire
BADS-SF: Behavioral Activation for Depression Scale–Short Form
BS: Beliefs Scale
CBT: Cognitive behavioral therapy
CESD-R: Center for Epidemiologic Studies Depression Scale–Revised
DAS: Dysfunctional Attitudes Scale
DAS-SF: Dysfunctional Attitudes Scale–Short Form
DSM-IV-TR: *Diagnostic and Statistical Manual of Mental Disorders, Fourth Edition, Text Revision*
GDB: Global Disease Burden
PANAS: Positive and Negative Affect Scale
PHQ-9: Patient Health Questionnaire, 9-item version
REBT: Rational Emotive Behavior Therapy
SWL: Satisfaction with Life
WHO: World Health Organization
